# Physiological and Proteomic Analysis in Chloroplasts of *Solanum lycopersicum* L. under Silicon Efficiency and Salinity Stress

**DOI:** 10.3390/ijms151221803

**Published:** 2014-11-26

**Authors:** Sowbiya Muneer, Yoo Gyeong Park, Abinaya Manivannan, Prabhakaran Soundararajan, Byoung Ryong Jeong

**Affiliations:** 1 Division of Applied Life Science (BK21 Plus), Gyeongsang National University, Jinju 660-701, Korea; E-Mails: sobiyakhan126@gmail.com (S.M.); iuyiuy09@naver.com (Y.G.P.); abinayamanivannan@gmail.com (A.M.); prabhakaran.s.bioinfo@gmail.com (P.S.); 2 Institute of Agriculture & Life Science, Gyeongsang National University, Jinju 660-701, Korea; 3 Research Institute of Life Science, Gyeongsang National University, Jinju 660-701, Korea

**Keywords:** blue-native page, chloroplast proteome, photosynthetic metabolism, salinity stress, silicon supplementation, *Solanum lycopersicum*

## Abstract

Tomato plants often grow in saline environments in Mediterranean countries where salt accumulation in the soil is a major abiotic stress that limits its productivity. However, silicon (Si) supplementation has been reported to improve tolerance against several forms of abiotic stress. The primary aim of our study was to investigate, using comparative physiological and proteomic approaches, salinity stress in chloroplasts of tomato under silicon supplementation. Tomato seedlings (*Solanum lycopersicum* L.) were grown in nutrient media in the presence or absence of NaCl and supplemented with silicon for 5 days. Salinity stress caused oxidative damage, followed by a decrease in silicon concentrations in the leaves of the tomato plants. However, supplementation with silicon had an overall protective effect against this stress. The major physiological parameters measured in our studies including total chlorophyll and carotenoid content were largely decreased under salinity stress, but were recovered in the presence of silicon. Insufficient levels of net-photosynthesis, transpiration and stomatal conductance were also largely improved by silicon supplementation. Proteomics analysis of chloroplasts analyzed by 2D-BN-PAGE (second-dimensional blue native polyacrylamide-gel electrophoresis) revealed a high sensitivity of multiprotein complex proteins (MCPs) such as photosystems I (PSI) and II (PSII) to the presence of saline. A significant reduction in cytochrome *b6*/*f* and the ATP-synthase complex was also alleviated by silicon during salinity stress, while the complex forms of light harvesting complex trimers and monomers (LHCs) were rapidly up-regulated. Our results suggest that silicon plays an important role in moderating damage to chloroplasts and their metabolism in saline environments. We therefore hypothesize that tomato plants have a greater capacity for tolerating saline stress through the improvement of photosynthetic metabolism and chloroplast proteome expression after silicon supplementation.

## 1. Introduction

Silicon (Si) is the most abundant element present in most soils, after oxygen. It plays an important role in plants at the bio-macromolecular level [1,2,3], and is easily taken up by a wide range of organisms due to its high solubility [4,5,6]. Silicon is a non-essential element, but its biological significance has been demonstrated in many species [2,7,8]. It is taken up by plants via their roots in the form of silicic acid (Si(OH)_4_) through silicon transporters and accumulates in the epidermis of various tissues, mainly as a polymer of hydrated amorphous silica. It has been observed that the transportation of Si from an external medium is mediated from cortical cells to the xylem by silicon transporter genes such as *Lsi-1* and *Lsi-2* [1]. Si plays an important role in molecular biology [3], and can contribute to the chemical defense of plants and their structural architecture [9]. Several studies have shown that Si treatment improves the growth and yield of various plants, particularly when they are subjected to either abiotic or biotic stresses [10,11,12,13,14]. However, at the chloroplast proteome level, no information has been published regarding how chloroplast multiprotein complex proteins interact with Si, nevertheless chloroplasts are the main targets for the production of reactive oxygen species during abiotic stress.

Salinity (NaCl) stress is one of the most significant abiotic stresses to agricultural crop production throughout the world [15,16,17]. Salt ions (NaCl) are readily absorbed by plants during water uptake, and minerals from soil or groundwater [18] in the xylem affects the composition of solutes, as well as electrochemical gradients and the proper function of transport systems [19]. Salt stress (NaCl) leads to a variety of metabolic changes in higher plants, such as reductions in photosynthesis and respiration, osmotic imbalances, ion toxicity, oxidative damage, and nutrient deficiencies [20,21,22,23]. Salt stress often exacerbates the generation of reactive oxygen species (ROS) [24,25,26], and ROS produced by such conditions may be extremely harmful to plants due to their ability to oxidize proteins, pigments, lipids, and nucleic acids, ultimately leading to the alteration of cell structure and mutagenesis [5]. The plant organelles specifically chloroplasts are sensitive to salt stress due to the generation of ROS. However, the specific mechanisms responsible for resistance to salt stress in chloroplasts, predominantly by Si supplementation, remain unknown. Of the plant organelles, chloroplasts are a prime target for stress inducers and can heavily influence chlorophyll content, photochemical quantum yields of photosynthesis [15], and light-harvesting complexes (LHC) I and II [26]. Salinity strongly inhibits the synthesis of chlorophyll [27] and their effective binding to proteins, thus reducing the accumulation of pigment-lipoprotein complexes, including photosystems I (PSI) [26] and II (PSII) [28]. A severe reduction in the content of large and small subunits of ribulose-1,5-bisphosphate carboxylase/oxygenase (RuBisCO), as well as other enzymes required for photosynthesis and chlorophyll biosynthesis have been observed under saline conditions [29,30]. Improvements in salt stress due to Si supplementation have been reported in several plant species such as wheat [31], barley [32], maize [33,34] and tomatoes [4] for various physiological processes although the effects on the chloroplast proteome are still largely unclear.

**Figure 1:**
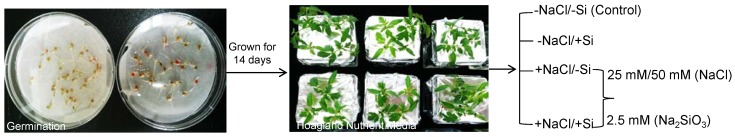
Demonstration plan used for designing nutrient, NaCl (sodium chloride) and Si (silicon) treatment used to supply to *Solanum lycopersicum* L. Seeds were germinated on filter paper in petriplates for 2–3 days under anaerobic conditions and later transferred to a 300 mL magenta box supplied with or without Silicon (Si) and NaCl in Hoagland nutrient media. Sodium chloride (25 and 50 mM) was prepared in +Si or −Si nutrient media to which plants were exposed for 5 days before harvesting and used in studies.

The primary objective of the present study was to investigate the strategies adopted by tomato plants when coping with salinity stress during silicon supplementation, with particular regards to the chloroplast proteome. The selection of tomato for our study was based on its importance as an important greenhouse crop in arid and semi-arid regions of Mediterranean countries, where soil and groundwater salinity are major problems that reduce tomato yield and quality [35]. We hypothesize that interactions between Si and NaCl (combined treatments shown in Figure 1) can alleviate the detrimental effects of salt stress on photosynthetic mechanisms. To test our hypothesis, composition of multiprotein complex proteins resolved with highly sophisticated techniques [36,37] by two-dimensional gel electrophoresis (2DE) followed by BN-PAGE (blue native polyacrylamide-gel electrophoresis) in the first dimension and SDS (sodium dodecyl sulfate)-PAGE in the second dimension were used. The proteomic map of a thylakoid proteome was scanned and images were analyzed for qualitative changes using MALDI-TOF/TOF-MS (matrix-assisted laser desorption/ionization time of flight mass spectrometry) coupled with MASCOT software to match the identified protein database. The content of photosynthetic pigments (total chlorophyll and carotenoid content), net-photosynthesis, transpiration and stomatal conductance were analyzed. Finally, stress markers in the form thiobarbituric acid reactive substances (TBARS), histochemical localizations of H_2_O_2_ and O_2_^−1^, concentrations of silicon (Si), sodium ions (Na^+^), and the biomass of plants were also investigated.

## 2. Results

### 2.1. Shoot Biomass Analysis

**Figure 2:**
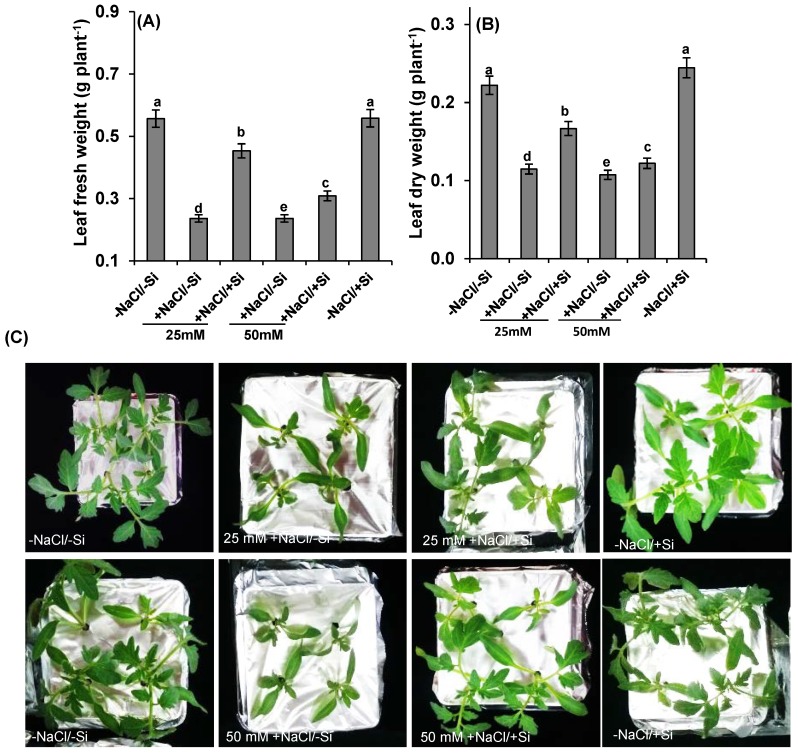
Fresh (**A**) dry weight (**B**) and morphological evidence (**C**) in leaves of tomato (*Solanum lycopersicum* L.) as affected by NaCl/Si combined treatments along with Control [−NaCl/−Si]: −Si with NaCl [25 mM, +NaCl/−Si], +Si with NaCl [25 mM, +NaCl/+Si], −Si with NaCl [50 mM, +NaCl/−Si], +Si with NaCl [50 mM, +NaCl/+Si] and with additional control +Si without NaCl [−NaCl/+Si]. Vertical bars indicate ± S.E. of the means for *n* = 5. Means denoted by the different letter are significantly different at *p* < 0.05 according to the Tukey’s studentized range test.

Salinity stress caused a significant decrease in shoot fresh and dry weight (Figure 2A–C). The fresh and dry biomass accumulation of salinity-treated tomato seedlings was significantly lower than plants that were adequately supplemented with Si (+NaCl/+Si) by 42%–58%. The greatest effect was observed significantly at 50 mM salinity stress by 60%–70% (+NaCl/−Si).

### 2.2. Silicon and Na^+^ Concentration

**Figure 3:**
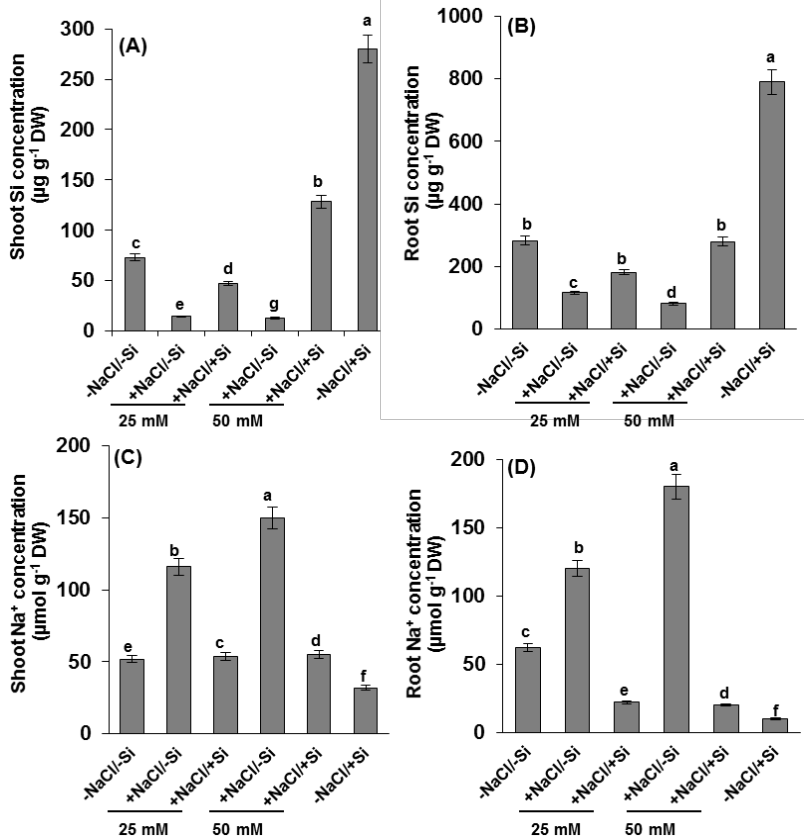
Changes in concentration of (**A**,**B**) silicon (Si) and (**C**,**D**) Na^+^ ions in roots and shoots of tomato (*Solanum lycopersicum* L.) as affected by NaCl/Si combined treatments along with Control [−NaCl/−Si]: −Si with NaCl [25 mM, +NaCl/−Si], +Si with NaCl [25 mM, +NaCl/+Si], −Si with NaCl [50 mM, +NaCl/−Si], +Si with NaCl [50 mM, +NaCl/+Si] and with additional control +Si without NaCl [−NaCl/+Si]. Vertical bars indicate ± S.E. of the means for *n* = 5. Means denoted by the different letter are significantly different at *p* < 0.05 according to the Tukey’s studentized range test.

Silicon (Si) concentration in shoots and roots of young tomato seedlings were observed in higher amounts approximately 25% more in plants grown under 50 mM (+NaCl/+Si) compared to 25 mM of salinity stressed under silicon supplements (Figure 3A,B). A significant differences in shoot silicon concentrations were observed under salinity stress (+NaCl/−Si) by 80%–90% when compared to control plants. The concentration of Si was observed highest in plants supplemented with Si alone compared to control plants.

To understand whether enhancement of growth by Si supplement is due to greater tissue tolerance under salinity stress or due to diminished uptake of NaCl, the concentration of Na^+^ ions were observed in roots and shoots of tomato plants (Figure 3C,D). The highest Na^+^ concentration, approximately 90% more compared to control (−NaCl/−Si), was observed in plants under 50 mM NaCl, while the lowest concentration was observed in plants which were supplemented with Si alone (positive control).

### 2.3. Oxidative Damage

**Figure 4:**
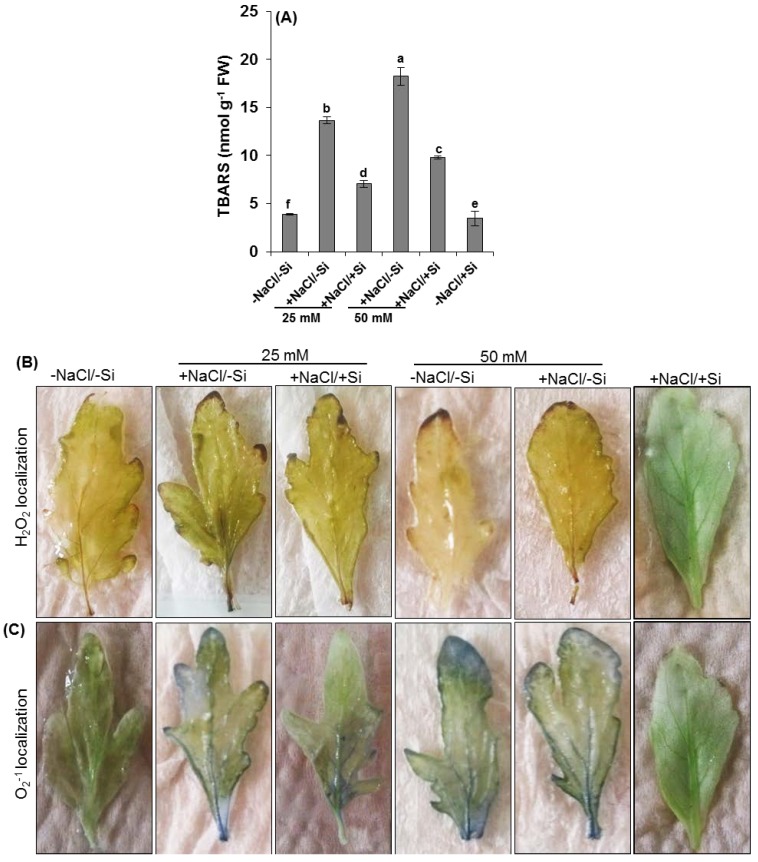
Oxidative damage marker in the form of (**A**) TBARS and histochemical localizations of (**B**) H_2_O_2_ and (**C**) O_2_^−1^ in leaves of tomato (*Solanum lycopersicum* L.) as affected by NaCl/Si combined treatments along with Control [−NaCl/−Si]: −Si with NaCl [25 mM, +NaCl/−Si], +Si with NaCl [25 mM, +NaCl/+Si], −Si with NaCl [50 mM, +NaCl/−Si], +Si with NaCl [50 mM, +NaCl/+Si] and with additional control +Si without NaCl [−NaCl/+Si]. Vertical bars indicate ± S.E. of the means for *n* = 5. Means denoted by the different letter are significantly different at *p* < 0.05 according to the Tukey’s studentized range test.

Oxidative damage was observed in NaCl/Si joint treatments as thiobarbituric acid reactive substances (TBARS). For the 25 mM salinity stress (+NaCl/−Si), levels of TBARS significantly increased in salinity stressed shoots (−25%), whereas higher increments of more than 90% compared to control, were observed in 50 mM of salt stressed plants (+Fe/−Cd) (Figure 4A).

Salinity stress (50 mM NaCl) treated leaves exhibited highly enhanced brownish staining compared to 25 mM NaCl (Figure 4B). The staining was not increased in the +NaCl/+Si leaves compared to controls. Production of O_2_^−1^ was studied by a reaction with nitro-blue tetrazolium (NBT), that is reduced by O_2_^−1^ giving rise to dark-blue spots of blue formazan. In both the 25 and 50 mM NaCl stressed leaves, dark blue spotted areas were widespread (Figure 4C) although a slight difference was seen between controls and +NaCl/+Si leaves.

### 2.4. Pigment Concentration

**Figure 5:**
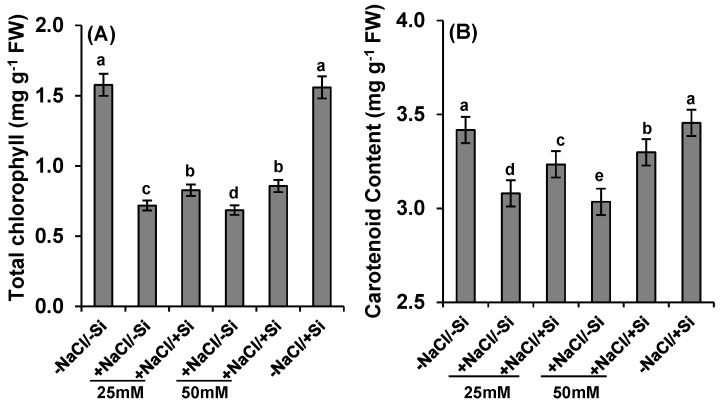
Changes in photosynthetic pigments (**A**) total chlorophyll and (**B**) carotenoid in leaves of tomato (*Solanum lycopersicum* L.) as affected by NaCl/Si combined treatments along with Control [−NaCl/−Si]: −Si with NaCl [25 mM, +NaCl/−Si], +Si with NaCl [25 mM, +NaCl/+Si], −Si with NaCl [50 mM, +NaCl/−Si], +Si with NaCl [50 mM, +NaCl/+Si] and with additional control +Si without NaCl [−NaCl/+Si]. Vertical bars indicate ± S.E. of the means for *n* = 5. Means denoted by the different letter are significantly different at *p* < 0.05 according to the Tukey’s studentized range test.

At 25 mM of salinity stress (+NaCl/−Si) in absence of silicon, total chlorophyll content decreased to 54.7% and was more reduced at 50 mM of salinity stress by 56% (+NaCl/−Si) (Figure 5A). This effect was significantly condensed when silicon (Si) was supplied, even in the presence of salinity stress (+NaCl/+Si).

Similarly, carotenoid content was decreased only by 9% at 25 mM of salinity stress (+NaCl/−Si) in the absence of silicon. This reduction was observed more by 11% at 50 mM of salinity stress, but these negative effects were amended by silicon supplementation (+NaCl/+Si) (Figure 5B).

### 2.5. Photosynthetic Measurements

**Figure 6:**
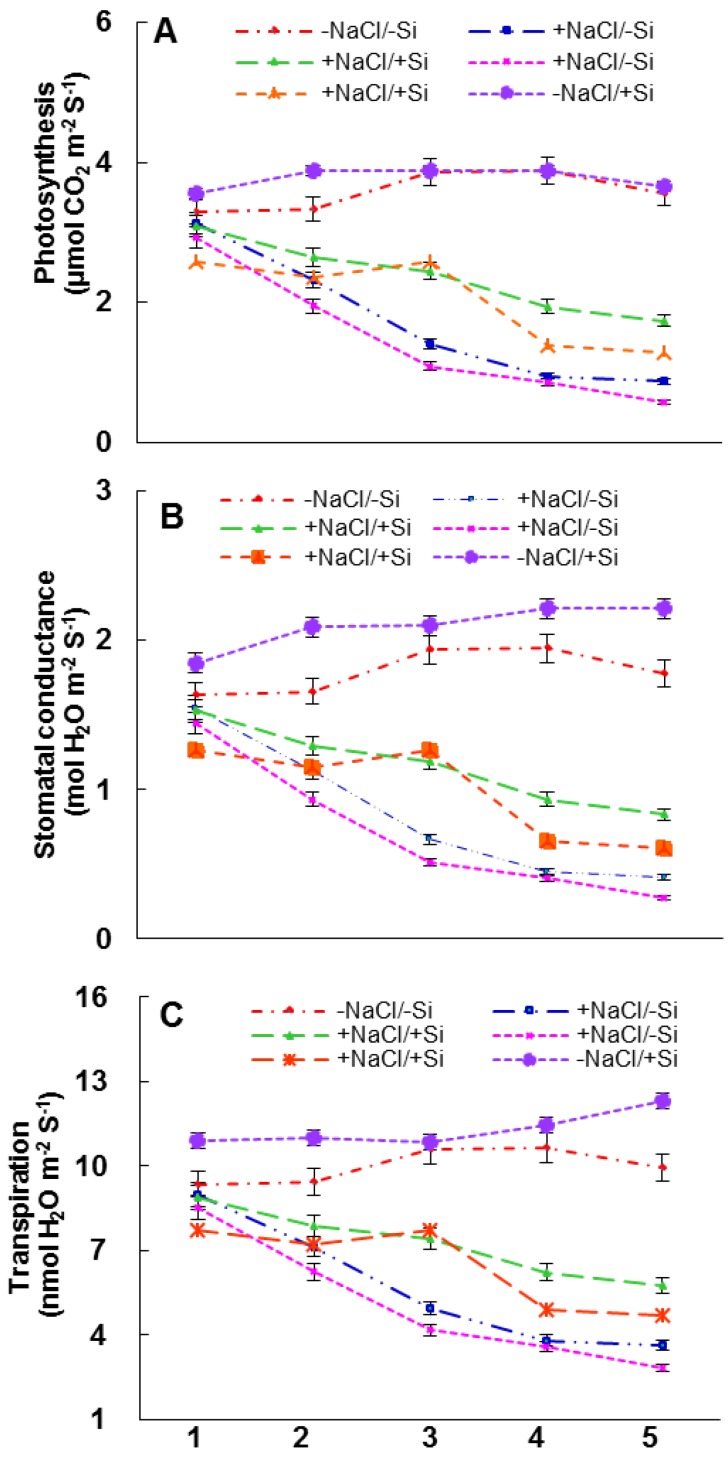
Changes in photosynthetic parameters (**A**) photosynthesis rate (**B**) stomatal conductance and (**C**) transpiration in leaves of tomato (*Solanum lycopersicum* L.) as affected by NaCl/Si combined treatments along with Control [−NaCl/−Si]: −Si with NaCl [25 mM, +NaCl/−Si], +Si with NaCl [25 mM, +NaCl/+Si], −Si with NaCl [50 mM, +NaCl/−Si], +Si with NaCl [50 mM, +NaCl/+Si] and with additional control +Si without NaCl [−NaCl/+Si]. Vertical bars indicate ± S.E. of the means for *n* = 5. Means denoted by the different letter are significantly different at *p* < 0.05 according to the Tukey’s studentized range test.

The photosynthesis rate was decreased by 25 mM of salinity stress (Figure 6A). The extent of the decrease was most prominent in the 50 mM salinity stressed leaves. The photosynthetic rate was improved to 66.2% of control in the presence of Si (+NaCl/+Si). The pattern of stomatal conductance (Figure 6B) and transpiration (Figure 6C) in response to Silicon and/or salinity stress were similar with that of photosynthesis rate. 

### 2.6. 1D BN-PAGE (First Dimensional Blue-Native Polyacrylamide Gel Electrophoresis) 

**Figure 7:**
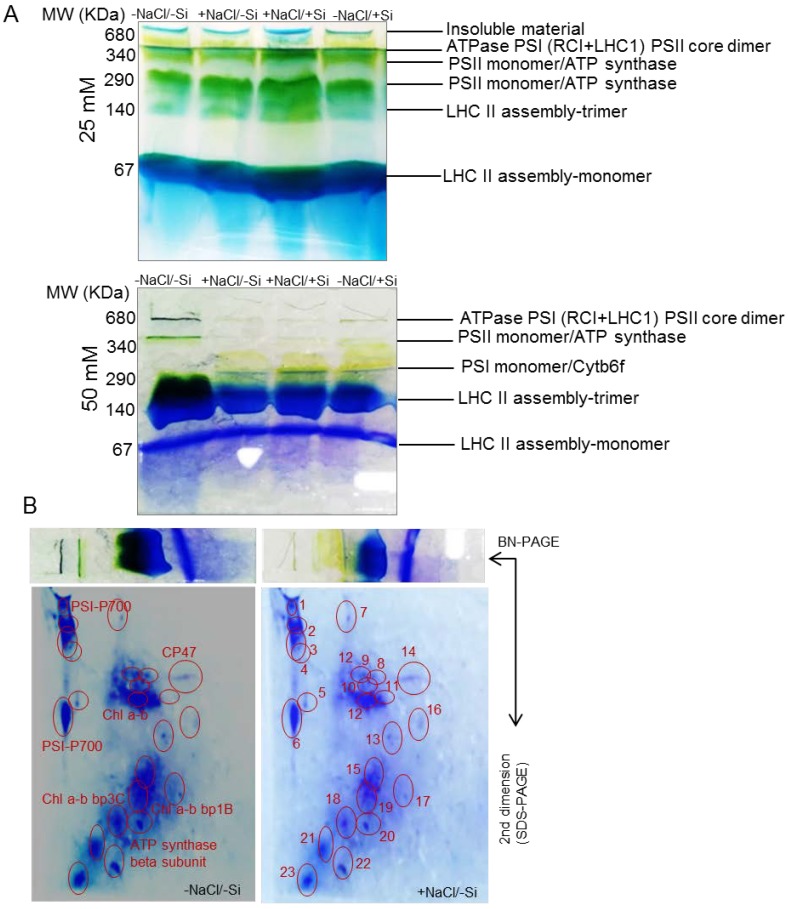
(**A**) First dimension BN-PAGE (**B**) 2D-SDS-PAGE in leaves of tomato (*Solanum lycopersicum* L.) as affected by NaCl/Si combined treatments along with Control [−NaCl/−Si]: −Si with NaCl [25 mM, +NaCl/−Si], +Si with NaCl [25 mM, +NaCl/+Si], −Si with NaCl [50 mM, +NaCl/−Si], +Si with NaCl [50 mM, +NaCl/+Si] and with additional control +Si without NaCl [−NaCl/+Si]. For first dimension BN-PAGE fresh thylakoid membranes from young leaves were solubilized in 1% BDM at chlorophyll concentration of 1µg·µL^−1^, and the protein sample was separated by 7%–12.5% gradient BN-PAGE. For second dimension gels slices were horizontally laid on top of 12.5% SDS-PAGE and stained with comassie brilliant blue R-250. Protein identification was based on previous reports and confirmed by MALDI-TOF/TOF-MS (shown in Table 1).

First dimensional electrophoresis run under native conditions on BN-PAGE was used to separate intact multiprotein complex proteins (MCPs) from thylakoids that were isolated from young tomato leaves. The leaves from a tomato were obtained after different treatment sets with three biological replicates. Figure 7A comprises the BN gel profile of thylakoids MCPs extracted from leaves under diverse treatments: (I) −NaCl/−Si (control) (II) +NaCl/−Si (25 or/and 50 mM) (III) +NaCl/+Si (25 or/and 50 mM) and (IV) −NaCl/+Si (positive control).

**Table 1:** Proteins identified in 2D-BN-SDS-PAGE map of tomato thylakoids.

Spot No.	Protein Identification	Plant Species	MASCOT Ion Score	NCBI Accession Number	Peptides	Sequence Coverage (%)
1	Self-incompatibility ribonuclease	*Solanum peruvianum*	32	K4AZ78	K.RWPQLK.H	12
2	Photosystem I P700 chlorophyll a apoprotein A2	*Solanum lycopersicum*	29	Q2MIA1	R.TPLANLIR.W	18
3	Non-specific lipid-transfer protein	*Solanum lycopersicum*	36	K4CLX6	MTFSKMQK.I	26
4	CEL4 = CELLULASE 4	*Solanum lycopersicum*	47	Q9S948	R.GSYSNGLRK.V	68
5	Glucose-6-phosphate isomerase	*Solanum lycopersicum*	33	K4BU47	K.SGGTPETR.N	63
6	Photosystem I P700 chlorophyll a apoprotein A2	*Solanum lycopersicum*	61	Q2MIA1	R.TPLANLIR.W	26
7	Uncharacterized protein	*Solanum lycopersicum*	42	K4CNV4	R.HEATQER.D	62
8	Oxygen-evolving enhancer protein 1, chloroplastic	*Solanum lycopersicum*	74	P23322	R.VPFLFTIK.Q	39
9	Oxygen-evolving enhancer protein 1, chloroplastic	*Solanum lycopersicum*	68	P23322	R.VPFLFTIK.Q	41
10	Oxygen-evolving enhancer protein 1, chloroplastic	*Solanum lycopersicum*	288	P23322	R.GSSFLDPK.G	58
11	Photosystem I P700 chlorophyll a apoprotein A2	*Solanum lycopersicum*	69	Q2MIA1	R.TPLANLIR.W	28
12	Chlorophyll a-b binding protein 1B	*Solanum lycopersicum*	50	P07370	K.FGEAVWFK.A	20
13	Chlorophyll a-b binding protein 1B	*Solanum lycopersicum*	46	P07370	K.FGEAVWFK.A	36
14	Photosystem II CP47 chlorophyll apoprotein	*Solanum lycopersicum*	38	Q2MI75	R.TGKPSLDLPK.I	36
15	Chlorophyll a-b binding protein 3C, chloroplastic	*Solanum lycopersicum*	37	P07369	K.FGEAVWFK.A	36
16	Photosystem II CP47 chlorophyll apoprotein	*Solanum lycopersicum*	57	Q2MI75	R.TPLANLIR.W	71
17	Chloroplast sedoheptulose-1,7-bisphosphatase	*Solanum lycopersicum*	44	C5IU71	K.VVDLLAPYR.R	71
18	Chlorophyll a-b binding protein 3C, chloroplastic	*Solanum lycopersicum*	69	P07369	K.FGEAVWFK.A	71
19	Chlorophyll a-b binding protein 1B, chloroplastic	*Solanum lycopersicum*	57	P07370	K.VVDLLAPYR.R	71
20	Photosystem I P700 chlorophyll a apoprotein A2	*Solanum lycopersicum*	61	Q2MIA1	R.TPLANLIR.W	26
21	ATP synthase subunit beta, chloroplastic	*Solanum lycopersicum*	57	Q9SCB5	K.VVDLLAPYR.R	23
22	Chlorophyll a-b binding protein 3C, chloroplastic	*Solanum lycopersicum*	69	P07369	K.FGEAVWFK.A	41
23	Chlorophyll a-b binding protein 1B, chloroplastic	*Solanum lycopersicum*	69	P07370	K.FGEAVWFK.A	41

A semi-quantitative analysis was performed for a comparison of the relative abundance of MCPs among all the treatments in each band of BN-PAGE. The identification of gel digestion of separate bands is listed in Figure 7B. The gel portions between 1000–680 KDa comprised of three super complexes of PSI-PSII were observed in lower quantity in salinity stress conditions (+NaCl/−Si), since these bands were expressed in higher amounts quantitatively in silicon supplemented tomato leaves. The dark green band at 340 KDa was observed as a PSII-monomer/ATP synthase; the intensity of this band was observed lower in 25 mM of salinity stress conditions, and further lowest when salinity conditions went up to 50 mM. Conversely this effect was amended when silicon was supplied to salinity-stressed plants. The light green band at 290 KDa comprises PSI-monomer/cytochrome *b6f* (band 3). A reduction of PSI-monomer/cytochrome *b6f* was observed in salinity stressed plants (25 mM) which further decreased its expression when salinity increased up to 50 mM, but after the supplements of silicon to salinity stress plants, reduction of the PSI-monomer/cytb6f was less marked.

LHC trimers (band 4) at 140 KDa showed a very interesting trend besides the reduction of intensity of this band at salinity stress conditions (+NaCl/−Si); this band was expressed in higher amounts in silicon supplemented plants even to control levels.

The dark blue band at 67 KDa contains LHC-monomers (band 5); this band was almost similar in all treatment sets when compared to control levels. 

### 2.7. 2D BN-SDS-PAGE

The first dimension BN-PAGE showed the resolution of multiprotein complex proteins of thylakoids in the form of bands and was further supported by 2D-SDS-PAGE electrophoresis (representative images were only shown for two treatments in Figure 7B). The BN-PAGE gels were loaded on SDS-PAGE for further protein identification analysis (Figure 7B). Approximately twenty four protein spots were detected on 2D-SDS-PAGE and all the treatment sets were compared with control. The protein profile was compared with other 2D-BN-PAGE maps of other species to confirm the exact match of identified protein spots from our results. From the second dimension 2D-BN-PAGE a dramatic reduction of protein spots were observed under salinity stress (+NaCl/−Si) compared to control, PSI and PSII super complexes were completely lost with 50 mM of salinity stress (Figure 7B) but, the presence of silicon helped the tomato plants retain the protein complexes. For LHC monomers and trimers the protein spots in 2D-SDS-PAGE were reduced in number under salinity stress (+NaCl/−Si), which was improved by supply of silicon (+NaCl/+Si). Therefore, silicon seemed to greatly amend the negative effect of salinity stress both quantitatively and qualitatively on MCPs.

### 2.8. Relationships among Descriptive Parameters with Si Concentration

Linear correlations among descriptive parameters of photosynthetic activity were assessed using the values as affected by NaCl and Si combined treatments (Table 2). Si concentration was closely related (*p* < 0.001) with descriptive parameters of photosynthesis (net-photosynthetic rate, transpiration and stomatal conductance) in leaves under 50 mM of NaCl/−Si treatments. Each of these parameters was highly associated with Si concentration, whereas much lower correlation was observed between photosynthetic pigments (total chlorophyll and carotenoid).

**Table 2:** Linear correlations among the descriptive parameters with Si concentration. The values measured at 25 and 50 mM after NaCl/Si combined treatment was used for correlation analysis. The correlation coefficient (*r*) and significant differences are given; *n* = 16. * *p* ≤ 0.05; ** *p* ≤ 0.01; *** *p* ≤ 0.001.

Treatments		FW	DW	TBARS	CL	CR	NPR	TR	SC
Si	(25 mM)	0.768 **	0.354 *	−0.894 ***	0.031	0.068	0.536 *	0.516 *	0.219
	(50 mM)	0.568 *	0.903 ***	0.935 ***	0.219	0.042	0.943 ***	0.933 ***	0.951 ***

Si, silicon concentration in leaves; FW, fresh weight in leaves; DW, dry weight in leaves; TBARS, thiobarbituric acid reactive substances in leaves; CL, chlorophyll; CR, carotenoid; NPR, net photosynthetic rate; TR, transpiration; SC, stomatal conductance.

## 3. Discussion

Due to several positive effects of silicon, it has been a focus of plant biology. Besides the amendment of various pathogens and parasites, positive effects of silicon on abiotic stresses such as toxic metals, salinity stress equally exist. Although several studies have been reported on silicon with several abiotic stresses including salt stress [38,39,40], little information exists on physiological aspects for chloroplasts. Thus, the present study provides experimental evidences on interaction between silicon (Si) and salt stress (NaCl) in chloroplasts. The earliest possible symptoms observed under salinity stress were chlorosis and necrosis and reduction in biomass (Figure 2). A significant decrease in biomass was observed under salinity stress. This result indicated that salt stress leads to morphological and physiological alterations in cell walls, which in turn can manifest the water imbalance in plants, which have been already emphasized in several plants [41]. This inequity was recovered by supplements of silicon (Si) as by increasing the biomass of plants. The possible role of Si to increase biomass and to decrease leaf chlorosis was also observed in cadmium toxic plants [42,43].

The total concentration of silicon and Na^+^ was affected by the presence of salinity stress (Figure 3) even under lower concentrations (25 mM). In recent studies halophyte grass [17] showed higher concentration of Na^+^ under salinity stress. In agreement with our findings, salinity stress showed higher concentration of Na^+^ whereas, Si supplement amended the concentration of Na^+^ in salinity stressed plants. Similarly our results showed that silicon concentration increased in silicon sufficient while decreased in salinity stressed tomato plants as also observed in previous studies [4]. Silicon (Si) accumulation, however, varies from species to species, generally in plants such as tomato, cucumber, and rice. An enhanced accumulation of silicon in salinity stressed tomato plants might be due to deposition of Si on cell walls of roots which have an ability to reduce the translocation of salts to the shoots [34]. In particular, plants under salinity stress often face oxidative damage [44,45,46], a greater variation was also observed in our studies by observing production of reactive oxygen species (ROS) under salinity stress as indicated by thiobarbituric acid reactive substances (Figure 4). The production of ROS might be due to loss of activity of antioxidant enzymes as previously reported in many plants under various abiotic stresses [47,48,49]. This negative effect was, however, recovered by supplement of silicon (Si) by decreasing the concentration of oxidative damages as shown by our study. The reduction of oxidative damage due to silicon supplementations in saline plants explains a great impact of silicon in amending abiotic/biotic stress [50].

In addition salinity stress resulted in decline in photosynthetic pigments (total chlorophyll and carotenoids) (Figure 5). The loss of these photosynthetic pigments was accelerated by the presence of silicon (+NaCl/+Si). The reduction of carotenoids might be responsible for loss of chlorophyll as carotenoids are the main components to absorb light during photosynthesis, which is responsible to protect the plants from photo-damage [51]. Since photosynthetic pigments are solely responsible for photosynthesis besides light, the reduction in these pigments also attributed a severe change in stomatal behavior [52,53]. The stress conditions have often been shown to increase leaf stomatal density [52,53], however, the present results showed that severe chlorosis and necrosis under high saline conditions might have led to a reduction in stomatal density. The loss of stomatal density led to poor exchange of gases, which resulted in more severe loss of stomatal conductance and transpiration rate (Figure 6) under salinity conditions. However, the supply of silicon (Si) recovered this deleterious effect and emphasized the induction of photosynthetic measurements. Silicon thus provides a factor to induce photosynthesis by preclusion of oxidative damage inside the chloroplasts, which has often been observed in other plants under drought or cadmium stress [54]. 

**Figure 8:**
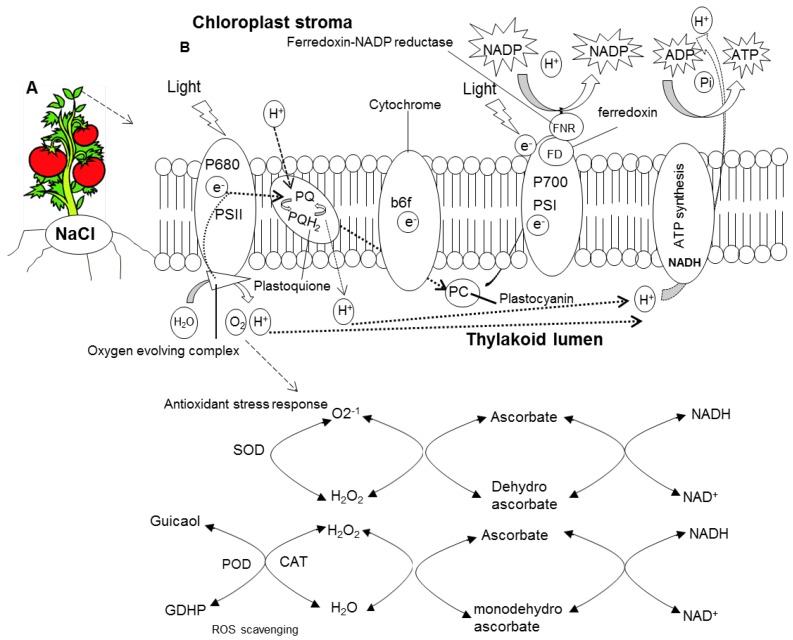
Schematic representation of a mechanism for production of oxidative damage in chloroplasts and its mitigation by the supplement of silicon (Si) (**A**) tomato plants exposed to salt stress (NaCl) (**B**) production of reactive oxygen species and defense mechanism in chloroplasts.

The decline in photosynthetic pigments in leaves may result in the overall decrease in bio-macromolecular chloroplast targeted proteins [50]. In recent years, several studies largely focused on physiological changes to silicon (Si) under abiotic stresses [55,56,57] including salinity stress. Less attention has been paid to the changes that occurred in light-dependent proteins of chloroplasts. Although there are several reports on changes in the chloroplast proteome under salinity stress, such as in tobacco leaves [58], soybean [45], rice [59] and mangroves [17], how to combat this change in the salinity-stressed chloroplast proteome has not yet been studied with silicon supplements. In our study first-dimensional BN-PAGE used as a proteomic tool to better-analyze chloroplast/thylakoid maps affected or executed upon salinity stress and/or further addition of silicon (Si) supply. In previous studies, such as in spinach, three dimensional electrophoresis was followed to meet co-migration of proteins [37], while as in case of *Brassica juncea* no co-migration was allowed [38] as also followed by tomato leaves in our studies. Plants exposed to constitutive NaCl considerably modified the composition of chloroplast protein complexes (Figure 7). Upon 50 mM of +NaCl/−Si treatment, strong reductions of PSI (RCI + LHCI), PSII, cytochrome *b6*/*f*, LHCII trimer complex were observed. The loss of most thylakoid protein complexes under salinity stress were largely improved in the presence of silicon (Si) (+NaCl/+Si), with LHC monomer as an exception. The consequent availability of NaCl thus limited the activity of chlorophyll *a*/*b* binding protein (PSI) however, this protein was observed up-regulated in salt-stressed halophytes [17]. The reduction of PSI in tomato plants suggested that it might reduce the excitation of energy given to reaction centers to increase NADP^+^ to NADPH generation, however, this was maintained to control level in silicon-supplied tomato plants. In this study, the combination of treatments caused synergic effects. It is well known that NaCl (salt stress) strongly disturbs the homeostasis of essential metal ions, which disturbs the proteome of chloroplasts. Our results demonstrated that in the presence of silicon (Si) plants were less damaged. This might be due to several factors such as competition of Si with NaCl for intake, the presence of sufficient amounts of silicon (Si) for assemblage of protein clusters etc. Thus, plants supplied with silicon (Si) helped plants to develop lesser amount of stress (ROS) in chloroplast under salinity stress. However, high concentration of salt (NaCl) caused non-significant and dramatic change to all photosystem complexes. Salinity stress caused overproduction of ROS and forced the chloroplast to destroy all protein components of chloroplast and forced antioxidant defense mechanism to stop its activity (see Figure 8 for ROS production and pathways for mitigation by Si supplements in chloroplasts). However, the supply of silicon (Si) overcomes these negative effects by combating the stress conditions.

This work shows that silicon (Si) helps tomato plants in keeping oxidative stress under control in chloroplasts/thylakoids. The quantity and quality of multiprotein complexes (MCPs) were recovered to a certain limit after addition of silicon in salinity stressed plants. Salinity is non-specific to plants to an extreme level and can be devastating to chloroplasts MCPs in several species, since it showed damage to photosynthetic components and loss of coordination and adverse effect on homeostasis whereas silicon can be influential to maintain photosynthesis even in the presence of salinity conditions as shown by our findings. Our results thus, indicate that Si supplementation plays a significant role in alleviating the chloroplast damage caused by salinity stress and that the damages are inversely intensified in the absence of Si in tomato plants.

## 4. Experimental Section

### 4.1. Plant Material and Treatments

Tomato seeds *Solanum lycopersicum* L. (Var. Dragonball, Korea) were washed with 1% sodium hypochlorite for 10 min followed by 3 washings in double distilled water (DDW). Sterilized seeds were germinated in the dark on wet filter paper in covered petri dishes for one week. The germinated seeds were then transferred in 300 mL magenta boxes to quarter-strength Hoagland nutrient solution for an additional 14 days containing (at mM concentrations for the macro elements): 1.0 NH_4_NO_3_; 0.4 KH_2_PO_4_; 3.0 CaCl_2_; 1.5 MgSO_4_; 0.15 K_2_HPO_4_; 0.2 Fe(III)-EDTA; and (µM for the micro elements): 14 H_3_BO_3_; 5.0 MnSO_4_∙H_2_O; 3.0 ZnSO_4_∙7H_2_O; 0.7 CuSO_4_∙5H_2_O; 0.7 (NH_4_)_6_MO_7_O_24_; 0.1 COCl_2_ in a controlled plant growth chamber with a 16 h photoperiod (under 100 µmol·m^−2^·s^−1^ light). A day/night temperature and relative humidity regimes of 25 ± 2 °C and 70% were implemented respectively. The nutrient solution was continuously aerated and renewed every 3 days. At the fifteenth day, young tomato seedlings were divided into different treatment groups consisting of: −Si/−NaCl [Control]; 25 mM salinity stress with no silicon [+NaCl/−Si]; 25 mM salinity stress with 2.5 mM silicon (Na_2_SiO_3_) [+NaCl/+Si]; 50 mM salinity stress with no silicon [+NaCl/−Si], 50 mM salinity stress with 2.5 mM silicon [+NaCl/+Si] and additional control consisting of 2.5 mM Si with no NaCl [−NaCl/+Si] (graphical representation for methodology is shown in Figure 1). The concentration of silicon supplement to salinity stressed plants were selected based on a previous report (Romero-Aranda *et al.* [4]). After 5 days of the treatment period, young plants were harvested and immediately frozen in liquid N_2_ before storage in a deep-freezer for further analysis. For biochemical and dry-biomass analysis the plants were oven dried at 70 °C for 48 h where necessary.

### 4.2. Assessment of Root Biomass

Plants were uprooted carefully from hydroponic culture media and softly blot-dried with lint-free paper. Each plant was separated into root and shoot material with the help of a sharp scalpel and forceps on moist paper sheets, which were then weighed for fresh biomass quantitation. 

### 4.3. Thiobarbituric Acid Reactive Substances (TBARS) Determination and H_2_O_2_ and O_2_^−1^ Localizations

Thiobarbituric acid reactive substances considered as oxidative damage products were determined by the method of Heath and Packer [60]. One gram of leaves were extracted in 0.1% trichloroacetic acid (TCA) (Sigma Aldrich, St. Louis, MO, USA), and centrifuged at 10,000× *g* for 5 min. The mixture containing 1 mL of supernatant with 0.5% thiobarbituric acid (TBA) (Sigma Aldrich) was heated at 95 °C for 30 min, cooled and centrifuged at 5000 rpm for 5 min. The absorbance of the supernatant was read at 532 nm and corrected for unspecific turbidity after subtraction from the value obtained at 600 nm.

To visualize H_2_O_2_ localization, leaves from all the treatments were immersed in a 1% solution of 3,3'-diaminobenzidine (DAB) (Sigma Aldrich) in Tris-HCl buffer (Promega, Fitchburg, WI, USA) (pH 6.5), vacuum-infiltrated for 5 min and then incubated at room temperature for 16 h in the absence of light. Leaves were illuminated until the appearance of brown spots characteristic of the reaction of DAB (3'3-diaminobenzidine) (Sigma Aldrich) with H_2_O_2_. Leaves were bleached by immersing in boiling ethanol to visualize the brown spots and were photographed with a digital camera.

For the visualization of O_2_^−1^, leaves were immersed in a 0.1% solution of nitro blue tetrazolium (NBT) (Sigma Aldrich) in K-phosphate buffer (pH 6.4), containing 10 mM Na-azide (Sigma Aldrich), and were vacuum-infiltrated for 5 min and illuminated until the appearance of dark blue spots (characteristic of blue formazan precipitate). After bleaching in boiling ethanol, the leaf samples were photographed as described above.

### 4.4. Estimation of Silicon and Na^+^ Content

For the determination of silicon (Si) and Na^+^ concentration, about 1 g of oven-dried leaf and root sample was digested with 50% perchloric acid (Promega) and concentrated H_2_SO_4_ (Promega) at 100–300 °C for 2–5 h respectively. The digested samples were then filtered with Whatman filter paper number 6 and diluted to a 100 mL by adding distilled water. The elemental content was determined by inductively coupled plasma optical emission spectrometry (ICP-OPTIMA 4300DV/5300DV/Perkin Elmer, Waltham, MA, USA).

### 4.5. Pigment Determination

Total chlorophyll and carotenoid contents were determined by dimethyl sulfoxide (DMSO) (Promega) as previously described by Hiscox and Israclstam [61]. Fresh leaves were collected in a glass vial to which 5 mL of DMSO were added and were kept in an oven at 65 °C (1 h) for complete leeching of pigments. The extracts were read by a UV-Vis spectrophotometer at 480, 645, 520 and 663 nm. The pigment concentrations in mg fresh samples were calculated using formulae given by Arnon [62].

### 4.6. Measurement of Photosynthetic Activity

Photosynthetic rate, stomatal conductance and transpiration levels were measured using a portable photosynthetic measurement system (LI-6400. LI-COR, Inc., Lincoln, NE, USA). The measurement was done 4 h after the beginning of the photoperiod under greenhouse conditions.

### 4.7. 2D BN-SDS-PAGE

BN-PAGE of integral thylakoid proteins was analysed according to Kügler *et al.* [63] with minor modifications. Thylakoid membrane were washed in washing buffer (330 mM sorbitol, 50 mM BisTris-HCl, pH 7.0) (Promega), and 0.1 mg·mL^−1^ pefabloc (Sigma Aldrich) as a protease inhibitor collected after centrifugation at 4500× *g* for 3 min at 4 °C, and the pellet was re-suspended in 25BTH20G (20% glycerol, 25 mM BisTris-HCl, pH 7.0 and 0.1 mg·mL^−1^ pefabloc) (Sigma Aldrich). An equal volume of resuspension buffer containing 2% *w*/*vn*-dodecyl-β-d-maltoside (Sigma, St. Louis, MO, USA) was added under continuous mixing on ice for 3 min for solubilization of membrane proteins. Insoluble material was removed by centrifugation at 18,000× *g* for 15 min. The resulting supernatant collected was mixed with 0.1 volume of loading dye (5% CBB-G250, 100 mM BisTris-HCl, pH 7.0, 30% *w*/*v* sucrose and 500 mM ɛ-amino-n-caproic acid) and were loaded on thick 1 mm 5%–12% *w*/*v* acrylamide gradient gel. For each sample, 100 µg of protein was loaded. Protein concentration was determined by Bradford method. The electrophoresis was performed at 4 °C in a Protean II xi Cell electrophoresis system (Bio-Rad, Hercules, CA, USA) for the first dimension by applying a constant voltage of 100 volts for 5–6 h and gradually increasing up to 200 volts until the run was observed complete. For protein separation in the second dimension the lanes from blue native gel were excised with the help of a sharp razor blade and incubated for 30 min at room temperature in SDS sample buffer containing 1% β-mercaptoethanol and sodium dodecyl sulphate (SDS) (Promega). The denatured BN lanes were then layered on 1 mm thick SDS-PAGE gels with 12% *w*/*v* acrylamide in the resolving gel. Proteins were separated at constant voltage of 100 using protean II xi cell electrophoresis system (Bio-Rad). The proteins were stained with comassie brilliant blue-R250.

### 4.8. Image Analysis

First and second dimension gel images were photographed using a high-resolution digital camera (Nikon, Tokyo, Japan). Image analysis was carried out with GelQuant.NET software (BiochemLabSolutions.com, University of California, San Francisco, CA, USA), which allowed band detection and quantification. 1D-BN-PAGE of each treatment was performed with three replicates (lanes). The normalization method provided by the GelQuant.NET software divides each lane by total sum of the total band volume to obtain individual relative band volumes. Total band intensity refers to the sum volume of all bands chosen for the analysis.

### 4.9. Protein in Gel Digestion and Identification by Matrix Assisted Laser Desorption/Ionization Time of Flight Mass Spectrometry (MALDI-TOF-TOF-MS)

The protein spots were excised manually with sharp razor from coomassie-stained gels, and in-gel digestion was performed by trypsin (Promega). The peptide mixtures were analysed with highly sophisticated matrix-assisted laser desorption/ionization time of flight mass spectrometry (MALDI-TOF/TOF-MS). The resulting mass spectra were used for protein identification by searching the database using MASCOT server (Matrix Science, www.matrixscience.com, London, UK). The searches were carried out using a mass window of 50–100 ppm for the precursor with monoisotopic mass accuracy, and fragment mass tolerance was ±0.6 Da. The search parameters allowed for carbamidomethylation (Cys), oxidation of methionine and allowed fixed modification. The positive identification was assigned with MASCOT scores with a score with other homology.

### 4.10. Statistical Analysis

A complete randomized design was utilized with five replicates. The Tukey’s studentized range test was employed to compare the means of separate replicates. Unless stated otherwise, the conclusions are predicated on differences between the means, with a significance level set at *p* < 0.05.

## 5. Conclusions

Our results thus, indicate that Si supplementation plays a significant role in alleviating the chloroplast damage caused by salinity stress and that the damages are inversely intensified in the absence of Si in tomato plants. Besides, silicon accumulation in plants is controlled by the ability of roots for its uptake However, the major drawback is that Si uptake in tomato plants is a complicated process because of its ability to accumulate low Si concentration compared to rice plants. The transport of Si is often controlled by *Lsi1* in subcellular localizations and has been already proved in tomato plants. The other two Si efflux genes such as *Lsi2* and *Lsi3* have possibility to exist in tomato and therefore, need to be characterized in tomato plants to overcome salt stress.
